# Cancer risk after radiotherapy for breast cancer

**DOI:** 10.1038/sj.bjc.6603235

**Published:** 2006-06-27

**Authors:** F Levi, L Randimbison, V-C Te, C La Vecchia

**Affiliations:** 1 Unité d'épidémiologie du cancer, Institut universitaire de médecine sociale et préventive, Bugnon 17, 1005, Lausanne, Switzerland; 2 Registres vaudois et neuchâtelois des tumeurs, Institut universitaire de médecine sociale et préventive, CHUV-Falaises 1, 1011, Lausanne, Switzerland; 3 Istituto di Biometria e Statistica Medica, Università degli Studi di Milano, Via Venezian 1, 20133, Milan, Italy; 4 Istituto di Ricerche Farmacologiche ‘Mario Negri’, Via Eritrea 62, 20157, Milan, Italy

**Keywords:** breast cancer, multiple tumours, radiation effects, radiotherapy

## Abstract

Among women with breast cancer, we compared the relative and absolute rates of subsequent cancers in 1541 women treated with radiotherapy (RT) to 4570 women not so treated (NRT), using all registered in the Swiss Vaud Cancer Registry in the period between 1978 and 1998, and followed up to December 2002. Standardised incidence ratios (SIRs) and the corresponding 95% confidence intervals (CIs) were based on age- and calendar year-specific incidence rates in the Vaud general population. There were 11 lung cancers in RT (SIR=1.40; 95% CI: 0.70–2.51) and 17 in NRT women (SIR=0.76; 95% CI: 0.44–1.22), 72 contralateral breast cancers in RT (SIR=1.85; 95% CI: 1.45–2.33) and 150 in NRT women (SIR=1.38; 95% CI: 1.16–1.61), and 90 other neoplasms in RT (SIR=1.37; 95% CI: 1.10–1.68) and 224 in NRT women (SIR=1.05; 95% CI: 0.91–1.19). Overall, there were 173 second neoplasms in RT women (SIR=1.54, 95% CI: 1.32–1.78) and 391 among NRT women (SIR=1.13, 95% CI: 1.02–1.25). The estimates were significantly heterogeneous. After 15 years, 20% of RT cases *vs* 16% of NRT cases had developed a second neoplasm. The appreciable excess risk of subsequent neoplasms after RT for breast cancer must be weighed against the approximately 5% reduction of breast cancer mortality at 15 years after RT.

Women treated with radiotherapy for breast cancer have an excess risk of lung, oesophageal, skin cancers and soft tissue sarcomas. Likewise, cohort studies based on cancer registries in Sweden (Prochazka *et al*, 2002), Switzerland (Levi *et al*, 2003), the UK (Roychoudhuri *et al*, 2004) and the US Surveillance, Epidemiology and End Results (SEER) Programme (Darby *et al*, 2005) found an excess lung cancer risk, with overall relative risks (RRs) between 1.3 and 2.0, with a trend towards increasing absolute and RRs with passing time after breast cancer diagnosis. Data from the Thames Cancer Registry (Roychoudhuri *et al*, 2004), the US Surveillance, Epidemiology and End Results (SEER) Programme (Ahsan and Neugut, 1998; Zablotska *et al*, 2005) and the Vaud and Neuchâtel Cancer Registries (Levi *et al*, 2005) indicated that women who had received adjuvant radiation therapy (RT) for breast cancer had an approximately two-fold excess risk of oesophageal cancer 10 years or more after breast cancer diagnosis. An excess of soft tissue sarcomas after breast cancer has also been reported (Hartley *et al*, 1993; Levi *et al*, 2003); the data are less clear for contralateral breast cancer (Roychoudhuri *et al*, 2004).

An overview of 78 randomised trials covering 42 000 women found a significant excess of contralateral breast cancer (RR=1.18), lung cancer (RR=1.61), oesophageal cancer (RR=2.06), leukaemias (RR=1.71), soft tissue sarcomas (RR=2.34), as well as all cancers other than breast (RR=1.20) (Early Breast Cancer Trialists' Collaborative Group [EBCTCG], 2005).

We therefore compared the relative and absolute risks of subsequent cancers among women treated with radiation for breast cancer (RT) to that of those not treated (NRT), using data from the Vaud Cancer Registry.

## PATIENTS AND METHODS

We used the data set of the Vaud Cancer Registry, which covers incident cases of malignant neoplasms in the Canton, whose population, according to the 31 December 2000 census, was 620 294 inhabitants (Levi *et al*, 2001). After exclusion of 21 cases detected at autopsy, 16 at death, 126 by death certification alone, 573 followed up for <1 month and of synchronous cancers (i.e. within 1 month after the first primary; *n*=25), the present series comprised 1549 RT and 4570 NRT breast cancer cases, registered between 1978 and 1998. These 6119 women were followed up to the end of 2002 for second primary neoplasms, emigration or death, with a total of 47 995 person-years at risk.

Calculation of expected number of cases was based on site-, age- and calendar year-specific incidence rates of the whole population of the Canton Vaud, multiplied by the corresponding number of person-years at risk. The significance of the observed/expected ratios (standardised incidence ratios, SIRs) and the corresponding 95% confidence intervals (CIs) was based on the Poisson distribution.

## RESULTS

As shown in Table 1, there were 11 lung cancers in RT (SIR=1.40) and 17 in NRT women (SIR=0.76), 72 contralateral breast cancers in RT (SIR=1.85) and 150 in NRT women (SIR=1.38), and 90 other neoplasms in RT (SIR=1.37) and 224 in NRT women (SIR=1.05). Overall, there were 173 second neoplasms in RT women (SIR=1.54, 95% CI: 1.32–1.78) and 391 among NRT women (SIR=1.13, 95% CI: 1.02–1.25). The estimates were significantly heterogeneous. These ratio between RT-treated and untreated women was 1.84 for lung cancer, 1.34 for breast, 1.30 for all other neoplasms and 1.36 for all neoplasms combined.

**Figure 1:**
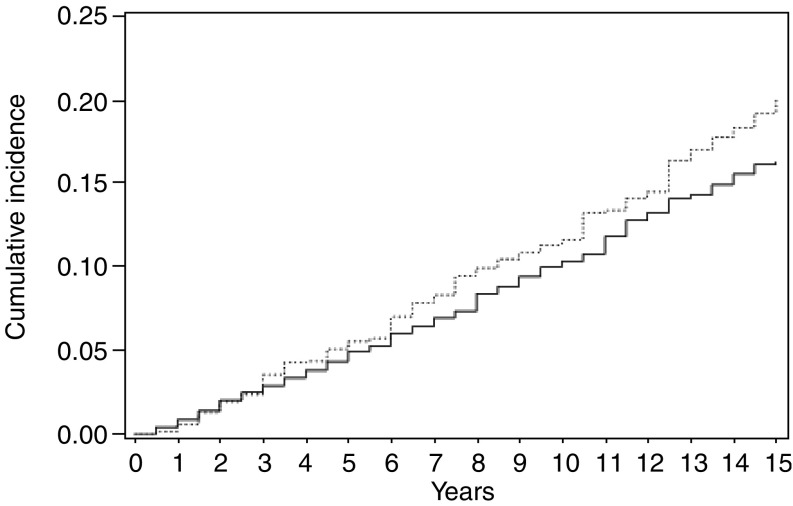
Cumulative rates for total cancer incidence in 1549 radiotherapy-treated (RT) and 4570 nonradiotherapy-treated (NRT) women up to 15 years after breast cancer diagnosis. Vaud, Switzerland. (-------------- RT; _________NRT).

Figure 1 gives the cumulative rates for total cancer incidence in the two treatment groups up to 15 years after breast cancer diagnosis. The rates started to diverge around 5 years after breast cancer diagnosis, when there were 5.5% in RT *vs* 4.9% in NRT women; at 10 years, these figures were 11.6% *vs* 10.3%, and at 15 years 20.2% *vs* 16.3%. The difference in cumulative incidence of second neoplasms was significant (*χ*^2^_(1)_=4.21, *P*=0.04).

## DISCUSSION

This study indicates that the risk of lung, breast, and also of all other neoplasms is increased in women who had received radiotherapy for breast cancer. The overall excess risk was about 30% for all neoplasms combined, comparable with those recorded by the EBCTCG (2005). The excess risk became evident in the first 5 years after radiotherapy, and after 15 years 20% of RT *vs* 16% of NRT women had developed a second neoplasm.

The present findings are broadly consistent with other population-based studies. Thus, the risk of second primary cancer was evaluated in a cohort of 525 527 women with breast cancer identified from 13 population-based registries in Europe, Canada, Australia and Singapore, and followed-up between 1943 and 2000 (Mellemkjaer *et al*, 2006). Among RT women, significant excesses were observed for cancers of the oesophagus, stomach, lung, soft tissue sarcomas, thyroid and leukaemias across subsequent strata of age and calendar years at first primary breast cancer. Similarly, in three cohorts (1975–1977, 1983–1985, 1991–1993) from the SEER Programme (Yu *et al*, 2006), at 8-year follow-up RT women had a significant excess risk ratio of 1.2 for cancers at all sites, as well as significant risk ratios for cancer of the breast (1.2), uterine corpus (1.3) and leukaemias (1.8).

Compared to the lung and breast, most sites probably receive considerably lower doses of radiation. However, the 30% excess risk observed for all other neoplasms is still compatible with the 0.97 excess RR per sievert for cohorts of workers occupationally exposed to low-dose ionising radiation (Cardis *et al*, 2005). Although the study is limited by the lack of data on tumour characteristics including nodal status, and of detailed information on type and dose of radiotherapy, and is unable to investigate the risk of the latest radiotherapy techniques, even reduced doses would still cause appreciable absolute risks of cancer.

The 4% excess absolute risk in total cancer incidence 15 years after RT has to be weighed against the 5% overall reduction in breast cancer mortality at 15 years (30.5% in RT *vs* 35.9% in NRT women) derived from an overview of 42 000 women from 78 randomised trials (EBCTCG, 2005). In the same overview, there was a 5.3% 15-year gain in total mortality in node-positive RT women after breast-conserving surgery, and a 4.4% gain in node-positive ones treated with mastectomy. However, for the overall data set of node-negative and positive women, the ratio of death rates for cancers other than breast cancer was 1.12 (EBCTCG, 2005).

In the SEER data set, the risk of radiation-induced heart disease was several times higher than the risk of radiation-induced second cancer (Darby *et al*, 2005). In the EBCTCG (2005), the excess mortality for circulatory disease was over two-fold higher than that for lung, oesophageal cancers, leukaemias and soft tissue sarcomas combined. Radiotherapy techniques have improved in recent years. Consequently, doses to organs other than the target have been reduced substantially over the last two decades. This is likely to have a major impact in reducing the excess mortality from heart disease, but its impact on cancer risk remains unquantified.

## Figures and Tables

**Table 1:** Number of observed second primary cancers of lung, breast and other primary sites among 1549 radiotherapy-treated (RT) and 4570 nonradiotherapy-treated women (NRT) diagnosed with first breast cancer between 1978 and 1998 and followed up to 2002, overall standardised incidence ratios (SIRs) and corresponding 95% confidence intervals (CIs), and ratios RT/NRT (Vaud, Switzerland)

	**Observed number**	**SIR (95% CI)**	**Ratio RT/NRT (95% CI)**
*Lung*			
RT	11	1.40 (0.70–2.51)	
NRT	17	0.76 (0.44–1.22)	1.84 (0.8–4.2)
			
*Breast*			
RT	72	1.85 (1.45–2.33)	
NRT	150	1.38 (1.16–1.61)	1.34 (1.0–1.8)
			
*Other second primaries*			
RT	90	1.37 (1.10–1.68)	
NRT	224	1.05 (0.91–1.19)	1.30 (1.0–1.7)
			
*All second primaries*			
RT	173	1.54 (1.32–1.78)	
NRT	391	1.13 (1.02–1.25)	1.36 (1.1–1.6)
